# Tackling the Temporal Stiffness of Kinetic Monte Carlo Simulations of Well-Mixed Chemical Systems via On-the-Fly Scaling and Cost-Error Optimization

**DOI:** 10.1021/acs.jpca.4c05963

**Published:** 2025-02-05

**Authors:** Giannis D. Savva, Michail Stamatakis

**Affiliations:** Thomas Young Centre and Department of Chemical Engineering, University College London, Roberts Building, Torrington Place, London WC1E 7JE, U.K.

## Abstract

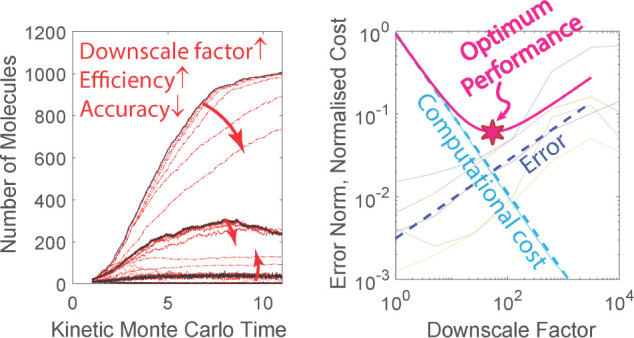

Reaction kinetics in biological systems are often subject to stochastic effects due to the low populations of reacting molecules, necessitating the adoption of kinetic Monte Carlo methods for their study. Such methods, however, can be computationally expensive, especially in the case of stiff systems, where some reactions are executed at much higher frequencies than others. We present an algorithm that reduces the reaction rate constants of the fast processes on-the-fly, thereby saving computational time, while keeping the approximation error within desirable limits. The algorithm couples the Modified Next Reaction Method for simulating stochastic systems with the Common Random Number framework and calculates accurate metrics for both the computational cost and approximation error by generating multiple sets of trajectories that correspond to increasingly reduced (downscaled) reaction rate constants. The optimum downscale factor is chosen via optimization of two conflicting objectives: (a) maximizing the speedup and (b) minimizing the approximation error introduced, and it is straightforward to tune the performance of the method, favoring accuracy versus speed or vice versa. Our approach is demonstrated on a biology-inspired well-mixed stiff system and is shown to accelerate the stochastic simulation thereof from 66 h down to 90 min, achieving a speed-up factor of 44×, without distorting the dynamics of the system studied.

## 1 Introduction

Kinetic Monte Carlo (KMC), and more specifically the Stochastic Simulation Algorithm (SSA), proposed by Gillespie,^1^ is a powerful computational tool that enables the study of interacting molecules and reaction intermediates in a homogeneous volume^2^ or over an explicitly defined surface (on-lattice KMC)^3^ while incorporating the inherent randomness of such systems. The trajectory produced using the SSA for a specific system is a sample path (realization) of the underlying Chemical Master Equation (CME),^4^ and in this sense, KMC is an exact method, but this benefit comes at an often significant computational cost. Thus, in systems with several species and multiple reactions, the time scales accessible by KMC might be too short to allow meaningful conclusions to be drawn. This issue is especially acute in systems in which some species exist in high populations. In addition, reactions may occur on different time scales, with some reactions being very frequent while others very rare. Since the reactions are executed one at a time, the KMC simulation would be dominated by the fast reactions, whereas the slow ones, which could be of interest and contribute to the evolution of the system, may not be executed enough times for adequate sampling. The scenario just described gives rise to an issue often termed “time scale disparity” or “stiffness” in a KMC simulation.

Multiple approximate acceleration methods have been developed throughout the years to tackle the time scale disparity in well-mixed chemical systems. A prominent such method is the τ-leap, developed by Gillespie,^5^ by which one “leaps over” many fast reactions in a single step and advances the KMC time accordingly, instead of executing all these events one at a time. The main assumption of the method is that the propensities of the fast reactions do not change significantly during the time leap. However, this assumption breaks down when species appear in small quantities, typically less than 10 molecules, which makes the method unusable for systems with low population species. Various refinements have been developed for the t^6,7^ address stability issues^8^ and prevent negative populations.^9−11^

Another approach to tackle time scale disparity is based on hybrid methods^12,13^ that combine the reaction rate equations with a discrete stochastic treatment of the system. The first step is to partition the reaction network into fast and slow reactions. Then, to reduce computational cost, the fast reactions are approximated in a deterministic way or via Langevin equations and the slow reactions are simulated as stochastic events using an implementation of the SSA.^12^ This approach achieves a bound on the computational cost by sacrificing accuracy, since fast fluctuations are approximated or eliminated, which might be acceptable depending on the nature and dynamics of the system studied.^12^ Building upon the previous work, Cao and coworkers developed a systematic approximate theory^14^ and then presented the Multiscale Stochastic Simulation Algorithm (MSSA)^15^ which assumes partial equilibrium for the fast reactions and works efficiently for low population species. Deterministically, partial equilibrium takes the form of algebraic constraints that the quasi-equilibrated species concentrations must always satisfy. Stochastically, partial equilibrium means that the probability distribution(s) over the corresponding states must be at quasi-steady state. However, the distribution over the partially equilibrated states is, in general, not known, and not easy to obtain.^15^

Other methods^16−20^ use perturbation analysis along with the quasi-steady-state approximation to reduce the CME, eliminate fast reactions from the reaction network and use the SSA to obtain realizations consistent with the reduced CME. This approach is shown to perform well in systems presented in the aforementioned studies. However, according to perturbation analysis, the reduced solution converges to the solution of the full model as the perturbation parameter, usually denoted by ε, approaches zero, or when it becomes “sufficiently small”. In general, ε is used to quantify the ratio of the slow versus the fast kinetics, and ε → 0 implies that the fast kinetics are much faster than the slow ones. In reality, ε is obtained by scaling appropriately the parameters of the model, and as a result, the order of magnitude of ε that ensures convergence depends on other parameters of the model. Another category of methods achieves computational savings by reducing appropriately the kinetic rates of frequent reactions in the course of KMC simulations.^21−26^ For example, the AS-KMC method,^21^ groups various frequently visited states into a superbasin whose barriers are raised, equivalently, their rate constants are reduced, in order to reduce their execution frequency, and therefore increase the probability that rare events occur. The above rate-reduction procedure includes certain parameters, whose values are chosen empirically. There is, however, an estimate of the error introduced by the rate-reduction procedure.

In practice, to tackle time scale disparity and reduce the computational cost of KMC simulations, one may manually reduce the rate constants of fast reactions in such a way that the dynamics of the system at the slow time scale remain unaffected. To achieve the latter, one usually performs a “convergence study” of a quantity of interest against the rate constant(s) that cause the chemical reaction system to become stiff. Initially, the rate constants of the fast reactions are aggressively reduced, aiming to distort the dynamics of the system. Then, the same rate constants are gradually increased (not to exceed the original values, though) until key indicators, such as the production rate of a species, have converged to a specific value. When convergence is observed, it is implied that further increase of the rate constants would have no effect on the dynamics of the system. Finally, the reaction rate constants for which the key indicators appear converged are used for production runs that are much cheaper computationally than using the original rate constants.

Similar to the approximate accelerated methods summarized above, the manual reduction of reaction rate constants does not provide any metric on the approximation error introduced by the reduction procedure. Although the procedure of reducing the rate constants of fast reversible reactions has physical meaning when the quasi-equilibration condition holds, there are no guarantees that the latter action has not incurred a change in the dynamics of the system under study. Aiming to address the lack of an accuracy index on the available methods, we have developed an on-the-fly downscaling scheme with an error metric that can be used to assess the effect of rate constants reduction on accuracy. Our method thus quantifies the actual computational cost and error of the simulation “on-the-fly”, by generating multiple trajectories with different rate constants. Based on this information, the rate constants of the fast reactions are reduced optimally, by taking into account the potential speedup gain while keeping the error introduced low. Overall, our method focuses on the optimization of the rate-reduction procedure so that the error introduced is within the user-defined limits while the computational savings are maximized.

The rest of the paper is organized as follows: the theory underpinning our approach and the implementation details of our on-the-fly rate constant scaling method are discussed in Section 2. The results obtained by employing our methodology to simulate a well-mixed stiff oscillatory system, along with a discussion on the performance and applicability thereof, are included in Section 3. Finally, Section 4 summarizes the motivation and main points of this work.

## 2 Methodology

### 2.1 Theory

Our algorithmic developments are based on an adiabatic approximation of the CME, in the presence of time-scale separation. We note that a clear-cut separation of time scales is an idealization that may not always be encountered in realistic situations, in which the simulated processes could span a wide range of overlapping time scales. However, we use the assumption of time-scale separation as a starting point for our development efforts, as it enables a rigorous mathematical analysis of the error of the approximation. Simulating systems with overlapping time scales can still be done with our algorithm by imposing a cutoff between fast and slow events and evaluating the actual error versus accuracy of the simulation on-the-fly, though this may lead to moderate performance gains (limited speedups).

We start by considering the following general master equation in a discrete space , with **x** and **x**′ denoting states therein ():
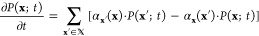
1

In the above,  denotes the propensity (probability per unit time) that the system will undergo a transition to state **x** in time *t* + d*t*, given that it was in state **x**′ at time *t*.

The presence of time-scale separation can be mathematically formulated by noting that the propensity of certain transitions takes large values, of the order of 1/ε, where ε is a small parameter, while the propensity of all other transitions is of the order of 1. Crucially, we assume that the fast versus slow transitions result in a hierarchical structure in the way states are connected in , and thus,  can be decomposed into subsets of states which undergo fast transitions. Denoting each of these subsets as , the statement just noted means that we can essentially introduce a change of variables, thereby representing each state **x** with an ordered pair (**u**,**v**), where **v** tells us which subset  the state belongs to and **u** is the index the state originally denoted by **x** within that subset. Then, the requirement that  is a set of states connected via fast transitions can be expressed as follows:
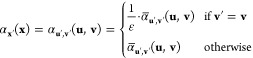
2

We highlight that, in the above, the scaled propensities  are of the order of 1. We also note that the probability that the system can be found in state **x** can now be expressed as
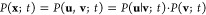
3where  is the conditional probability that the system is in state **u** given that it is within the subset . Then, the master equation can be rewritten as follows:
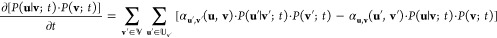
4

Exploiting the presence of a small parameter ε in the master equation, by virtue of eq 2, we can introduce asymptotic expansions with respect to ε for the probabilities as follows:
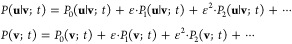
5

From the master eq 4, after using (2), (3), and (5), and collecting terms of order , we get:
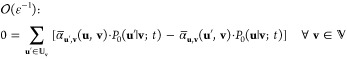
6

As the above is a set of algebraic equations, it is clear that  are no longer differential variables; they are now algebraic variables but in general are still nontrivial functions of time. Furthermore, repeating the procedure but now collecting terms of order :
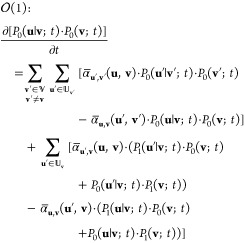
7

The presence of *P*_1_ in the above equation introduces a closure problem, which, however, can be resolved in a straightforward way by applying the operator  to both sides of the equation, thereby making the second (single) sum of the right-hand side vanish. Hence, eq 7 simplifies to
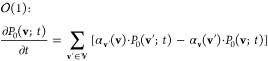
8where:
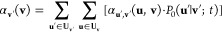
9

Clearly, , since transitions within the subset  conserve the conditional probability. Equations 8 and 9 reveal that under the assumptions of time-scale separation and the separable structure of the state space, as discussed above, the slow dynamics (transitions between the subsets ) are governed by a new master equation. In the latter, the propensities are weighted averages of the “original” state-to-state propensities, with weighting factors given by the conditional probability , obtained by the solution of eq 6. At infinite time scale separation (ε → 0), the set of eqs 6, 8 and 9 becomes exact, and thus, the trajectories of the slow variables, **v**, become invariant to the fast dynamics. This key result is the basis of our algorithmic implementation.

### 2.2 Conceptual Outline of the Algorithm

Motivated by the theoretical analysis just presented, it should be possible to generate and compare over a short KMC time interval two trajectory chunks, the first obtained by using the original rate constants, while the second generated using reduced (“downscaled”) rate constants for the fast events, which would clearly be computationally cheaper. If a comparison of all the interarrival times (IATs) of the slow reactions identifies no differences among the two trajectories, then the reduction in the rate constants did not distort the dynamics of the system. Therefore, one can continue propagating the system in time with the reduced rate constants instead of the original ones, benefiting from the lower computational cost of simulating the (now less frequent) fast events.

In this spirit, the purpose of our approach is to tackle the time scale disparity in well-mixed systems by reducing “on-the-fly” during the KMC simulation, the execution frequency of the very fast (extremely frequent) reactions so that the KMC simulation reaches far greater time scales. To achieve the invariance of the slow dynamics in a practical setting, we combine the Modified Next Reaction Method (Mod-NRM) for propagating the state of the system^27^ with state-of-the-art ideas adopted in sensitivity analysis studies, in particular, multiple random number streams as implemented in the Random Time Change (RTC) method^28^ as well as the Common Random Number (CRN) method.^29^

Crucially, our methodology is capable of quantifying the error introduced in the system because of the reduction of the rate constants. This is achieved by generating multiple trajectory chunks over a specific KMC time interval, with each one of them corresponding to progressively lower rates of the fast reactions, obtained by rate constants that are downscaled by an increasing factor. We quantify both computational cost and error, combine them in an objective function and solve an optimization problem to identify the downscale factor that provides an optimum balance between speed versus error. Favoring the former or the latter of the two conflicting objectives is achieved through a user-defined parameter in the objective function. In the following, we provide detailed descriptions of the components and procedures of our approach, while the already well-established underpinning methods are discussed in Section S1.

### 2.3 Algorithmic Details

Once the KMC simulation is up and running with the original reaction rate constants, the algorithm determines a KMC time interval, (*t*_*s*_, *t*_*f*_), *t*_*f*_ > *t*_*s*_, over which it is invoked, in order to evaluate the feasibility and correctness of a reduction in the rate constants of the fast reactions, and perform this downscaling if appropriate. Over that KMC time interval, (*t*_*s*_, *t*_*f*_), the algorithm generates multiple trajectories with rate constants reduced by an exponentially increasing downscale factor, such as 5, 25, 125, 625, etc. Since the downscale factor is applied only to the rate constants of the very frequent reactions that consume most of the computational time, the execution frequency thereof decreases proportionally to this factor; thus, the generation of each additional trajectory chunk is progressively less costly. The computational cost of each individual trajectory chunk is estimated by using the number of KMC steps executed over the aforementioned time interval (*t*_*s*_, *t*_*f*_).

While the computational cost decreases with a more aggressive downscaling, the accuracy of the simulation decreases, but quantifying this loss of accuracy is nontrivial due to the inherently stochastic nature of KMC simulations. Thus, we need a reliable procedure to discern changes in the system’s trajectory arising from (a) the reduction of the rate constants of the fast reactions versus (b) the inherent stochasticity of KMC. Aiming to eliminate stochasticity and focus on changes incurred solely because of the modification of the rate constants, we use the CRN technique^29^ (see also Section S1.5 for a brief summary of this technique). In addition, we make use of multiple random number streams, along the principles of the RTC method.^28^ More specifically, we generate the firing IATs for all the fast reaction channels by drawing random numbers from a single random number stream, whereas we use separate random number streams, one per reaction channel, for the slow reactions. Finally, we quantify the accuracy of the downscaled trajectories as compared to the reference one, in terms of the IAT differences of the slow reactions in a way that is described in detail further below.

By collecting information on the scaling of the computational cost, *C*, and error, *E*, with respect to the downscale factor, *df*, we compose the following objective function, *F*(*df*):
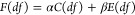
10with α and β, being the weights on the computational cost, *C,* and error, *E*, respectively. Given the objective function, the goal is to identify the optimal downscale factor, *df*_opt_, that maximizes the speed-up gain with the least error. Once that factor has been determined, the rate constants are modified accordingly, and the simulation continues propagating the system in time with the reduced rate constants. The above procedure is invoked regularly at specific KMC time intervals throughout the KMC run and aims at identifying whether further downscaling would be possible without increasing the error to unacceptable levels.

#### 2.3.1 Partitioning the Reaction Network into Fast and Slow Reactions

As our methodology uses a different random number stream for each one of the slow reaction channels, the correct identification of the reactions that are expected to be slow is a crucial first step toward setting up our algorithm, so as to initialize the correct number of independent random number streams. To classify the reaction channels into fast and slow, the user needs to first provide information on the ordering of the execution frequency of reactions, obtained from a short trial run. In practice, the user provides a ranking of the reaction channels from the fastest to the slowest and pinpoints those reactions whose rates are allowed to undergo downscaling. The downscalable reactions are restricted to forward–reverse pairs only in order to maintain detailed balance and quasi-equilibrium, and before invoking a downscaling, we check whether those reactions are indeed quasi-equilibrated (QE) using the criterion:^26^
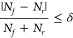
11where *N*_*f*_ and *N*_*r*_ is the number of executions of the forward and reverse reaction, respectively, and δ is a threshold value chosen as 0.05. It is worth noting that, certain situations are conceivable for which the equation just presented could incorrectly classify nonquasi-equilibrated events as QE. Snyder et al., for instance, have discussed distributed lattice-based KMC simulations with symmetric diffusion events, in which the initial and final states (a pair of sites one of which occupied and the other vacant) are related via rotation/reflection. In this case, the average number of forward and reverse events can be equal even though coverage gradients may exist, and thus the system may not be (quasi)-equilibrated. In such cases, one should consider alternative approaches to identify QE processes, e.g., in the case discussed by Snyder et al., one could partition the domain into subunits and compare the net diffusion rates across different subunits, in order to decide whether diffusion is quasi-equilibrated.

In any case, the above criterion (eq 11), or an appropriate alternative, along with the user-provided information on the expected ordering of the reaction frequencies are taken into account while attempting a downscaling. If this criterion fails, namely, the value of the ratio in eq 11 is greater than 5% or the ordering of execution frequencies changes, then the attempt is terminated and the KMC simulation continues with the most recent rate constants.

The classification of processes into fast and slow can, in principle, be done automatically. In the presence of nonreversible reactions however and especially in oscillatory systems,^30,31^ the latter procedure is not trivial and requires deeper analysis of the reaction network. Since our focus is to develop an on-the-fly rate scaling algorithm that optimizes cost and error, we opted for requesting user input on the reaction network and postponed the automation of the partitioning procedure for a future improved version of our algorithm.

#### 2.3.2 Generating Multiplicates with CRNs and Different Rate Constants

The assessment of the error introduced and the reduction in computational cost is based on comparing a reference KMC trajectory against multiple trajectories generated by the CRN method, using progressively reduced rate constants. In particular, following the initialization step, the KMC simulation starts as usual and after the execution of *N* KMC steps, at KMC time *t*_*s*_, our algorithm is invoked to check whether a reduction on the rate constants of the fast processes is feasible. At time *t*_*s*_, a snapshot is saved that contains information on the current state of the system such as the species populations, the rate constants of all reactions and the states of the random number generators that correspond to the slow reactions.

**Figure 1:**
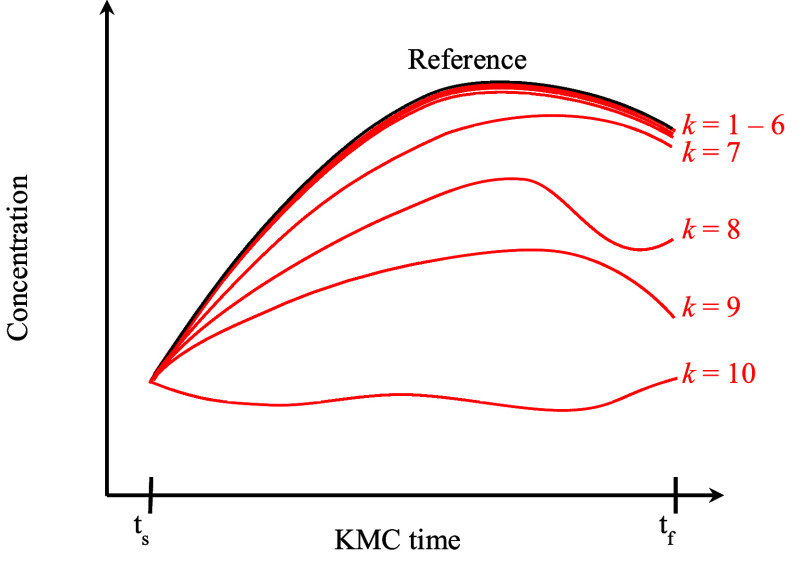
Schematic representation of multiple trajectories generated over the KMC time interval (*t*_*s*_, *t*_*f*_). All trajectories have the same starting point provided by the saved snapshot at time *t*_*s*_. Evidently, the system’s dynamics have definitely been distorted from the *k =* 7 downscaled trajectory onward.

Following the snapshot saving, the algorithm propagates the system until KMC time *t*_*f*_ with the same rate constants used up until *t*_*s*_, that is, the rate constants are not modified. The time *t*_*f*_ is chosen such that (a) propagating the system further for *w = t*_*f*_ – *t*_*s*_ KMC units is still tractable in a reasonable amount of time and (b) all reactions, especially the slow ones, are sufficiently sampled, although the latter is not strictly necessary for reasons that will become clear further below. In addition to the usual KMC procedures performed, such as species population sampling, saving into output files and incrementing reaction counters, over the interval (*t*_*s*_, *t*_*f*_) we record the occurrence times *t*_*i,j*_ of every single reaction firing *i* belonging to any slow reaction channel *j*. The generation of the trajectory with the unmodified rate constants is important since it serves as our reference in subsequent comparisons. We thus refer to this trajectory as the “unscaled” or “reference” trajectory. A schematic representation of one such trajectory for a species of interest is illustrated in Figure 1 by the black curve.

Once the unscaled trajectory is generated, we perform the checks previously described (see Section 2.3.1). At first, we check whether the fast reversible reactions, whose rates we are allowed to reduce, are quasi-equilibrated using the criterion (11). Second, we order the reaction frequencies from the fastest to the slowest and we compare their ordering against the expected ordering that the user provided. More specifically, we check if the anticipated fast reactions are indeed fast and the anticipated slow reactions are indeed slow in terms of their execution frequencies. The latter is a necessary check because in oscillatory systems the execution frequencies might not have the same ordering for any arbitrarily chosen KMC time interval. Third, we quantify the time scale separation, TSS, as the logarithm of the ratio of the execution frequencies among the fast and slow reactions. The purpose of the logarithm is to obtain the TSS in term of orders of magnitude. The user provides TSS_min_, the minimum time scale separation, which implies that if the fast reactions are not fast enough, no downscaling is attempted. The purpose of the time scale separation criterion is to prevent making the initially fast reaction too slow, or comparably slow to the initially slow ones. If any of the above checks fails, the unscaled trajectory is accepted as the “official” trajectory over the interval (*t*_*s*_, *t*_*f*_) and the simulation continues from KMC time *t*_*f*_. In the case where the above checks indeed show that the fast processes are quasi-equilibrated in the interval (*t*_*s*_, *t*_*f*_), their ordering is as expected, and the fast reactions are fast enough, the algorithm proceeds in generating the downscaled trajectories.

Having generated the unscaled trajectory, the algorithm restores the state of the system back at time *t*_*s*_ using the saved snapshot. The latter action restores the random number generators of the slow reactions to the state they had at *t*_*s*_. This is done intentionally, so that the same random numbers are used for the generation of the occurrence times for the slow reactions in the interval (*t*_*s*_, *t*_*f*_), in line with the CRN methodology.^28^ On the other hand, the occurrence times of the fast processes are not of interest and are thus not saved at all, neither is their generator’s state saved or restored. Then, the rate constants of the fast processes are reduced by a user-defined “base downscale factor”, *df*_b_, a downscaled trajectory is generated over the KMC time interval (*t*_*s*_, *t*_*f*_), and the occurrence times *t*_*i,j*_ of all slow reactions are recorded. Using the state snapshot at *t*_*s*_ as the starting point, a total of *k*_max_ downscaled trajectories are generated, each one with an exponentially increasing downscale factor of , where *k* is the index of the downscaled trajectory being generated, e.g., if , the first downscaled trajectory is generated by dividing the original rate constants by , the second by , the third by  and so on. The red curves of Figure 1 present schematically some of these downscaled trajectories accompanied by their index *k*. In our example the low-index downscaled trajectories 1 to 6 are close to the reference trajectory so they are displayed as a bundle in Figure 1. It is important to note here that during the generation of the first downscaled trajectory, corresponding to *k* = 1, the rate constants are actually not reduced since the downscale factor is one. Despite the additional computational cost, this trajectory, in combination with the unscaled trajectory, whose generation was described above, are both crucial in determining a baseline for the error of the calculations as we will see shortly.

At the end of the above procedure, we have obtained one unscaled and *k*_max_ downscaled trajectories, over the same KMC time interval (*t*_*s*_, *t*_*f*_) while reusing exactly the same random numbers for the occurrence of the slow reactions (Figure 1). In addition, we have obtained the absolute occurrence times of all the slow reaction firings along with the total KMC steps executed for every trajectory generated. The latter quantities are used to quantify the error and computational cost respectively, as discussed next.

#### 2.3.3 Cost and Error Quantification and Optimization

**Figure 2:**
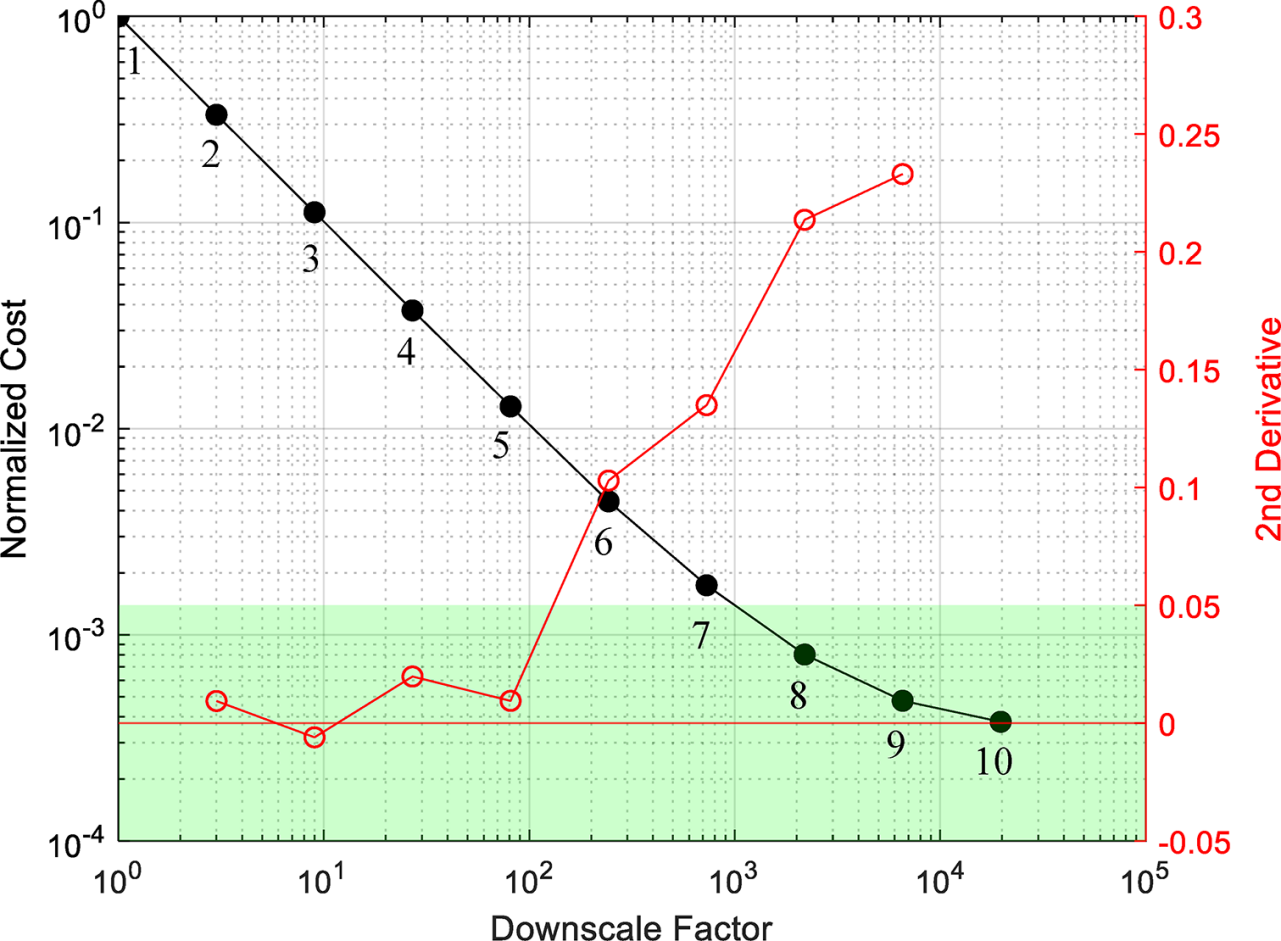
Black curve: normalized computational cost with respect to the downscale factor. Red curve: numerical 2nd derivative of the normalized cost data points with its values as given by the right *y*-axis. The green horizontal band represents the acceptable range for the values of the 2nd derivative.

A measure of the computational cost for the generation of each one of the alternative trajectories is the number of KMC steps executed. To derive a relative dimensionless cost for each trajectory we divide the total number of KMC steps executed therein by the number of KMC steps of the unscaled trajectory, so that the latter has a dimensionless cost of one. We then identify the maximum downscale factor that does not distort the dynamics of the system as follows. Since we are downscaling only the fast reactions, we expect the computational cost to drop via an inversely proportional relation with respect to the downscale factor: cost ∼ 1/d*f*. Hence, the normalized cost plotted on log–log axes with respect to the downscale factor would appear as a line with gradient of −1, as illustrated in Figure 2 from the first up to the sixth black data point.

The relationship just noted should hold true as long as the reactions whose rate constants are reduced are much faster than all other reactions and dominate the computational simulation. Violations of this condition may occur, and potentially indicate that the initially fast reactions are no longer the most time-consuming ones. To detect such violations and identify the downscale factor after which the cost does not decrease as expected, we use two different methods, thereby ensuring the robustness of the procedure.

First, we perform successive linear fittings on the log(cost) versus log(*df*) data using a sliding window approach with a set of just four data points each time, e.g., in Figure 2, the linear fit is performed using data points 1–4, 2–5, 3–6, etc. When the gradient of the linear fit is larger than −0.9, the second to last data point of the set is marked as the maximum valid downscale factor, *df*_max_1_. Second, we calculate the second derivative of our data points using central finite differences, as illustrated by the red curve in Figure 2. For a linear relation the second derivative is zero; thus, when the second derivative exceeds the threshold of 0.05, we have identified the maximum valid downscale factor, *df*_max_2_. The minimum among (*df*_max_1_, *df*_max_2_) is taken as the final *df*_max_ and we discard the data points for larger downscale factors as invalid. Next, to obtain an analytical expression for the cost, *C*(*df*) in eq 10, we fit a line to the above data points that are deemed valid.

We have so far discussed how we quantify the cost of the simulation, and we now move on to discuss the error. The main motivation in using Anderson’s Mod-NRM^27^ is that it decouples the randomness in the model from the state of the system. In addition, using different random number streams for each slow reaction channel makes the firing times of each channel independent from those of other channels. Restoring the states of the random number generators when generating a new downscaled trajectory ensures that the internal firing times are preserved across the different runs which is equivalent to keeping the reaction path of the slow processes the same, in line with the CRP method.^28^

Thus, to quantify the error incurred by downscaling, we use the occurrence times *t*_*i,j*_ to calculate the IATs τ_*i,j*_ for every single reaction firing *i* from the slow reaction channel *j* for the unscaled and all the downscaled trajectories. Then, for each downscaled trajectory *k*, and each slow reaction channel *j*, we calculate the IAT difference vector, **d**, with respect to the unscaled trajectory as
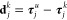
12where  is the vector holding the IATs of the unscaled trajectory, hence the superscript *u*. In general,  and  have the same number of elements, *i*_max_, which is equal to the number of reactions fired. If it happens that their sizes differ, because there was a different number of reactions fired in the unscaled, *u*, and in the downscaled trajectory with index *k*, the minimum number of elements is used, corresponding to the common reaction firings. Note that observing a different number of firings for a slow reaction channel *j* is more likely to occur at large downscale factors. Lastly, for each *j* we define the IAT error norm as the Euclidean norm of the IAT difference vector :
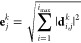
13

The above metric quantifies the difference between two trajectories in terms of their IATs differences. We calculate the error norm for every downscaled trajectory *k* thus obtaining the error scaling for a particular slow reaction channel *j* up to *df*_max_. To obtain an analytical expression for the scaling of the overall error, we fit the scaling equation  to all error norm data points, with *p* and *q* being parameters determined by the fitting, and *x, y* being the downscale factor and IAT error norm, respectively. This procedure is analogous to fitting a first-order polynomial to the logarithms of the error norm and downscale factor, just like in the case of the computational cost. We thus obtain the second component, *E*(*df*), of eq 10.

At this stage, using the user-provided weights on cost and error, along with the analytical expressions for the scaling of cost and error, *C*(*df*) and *E*(*df*), respectively, fitted to the data obtained from the KMC simulation, we form the objective function *F*(*df*), as per eq 10. By minimizing *F*(*df*), we identify the optimal downscale factor, *df*_opt_, to use for the current downscaling attempt. Using *df*_opt_, we generate a trajectory using rate constants reduced by *df*_opt_, until a KMC time of *t*_*f*_, and save the trajectory as the “official one”, along with the samples taken before *t*_*s*_.

Then at KMC time *t*_*f*_, the algorithm exits the downscale mode and continues propagating the system with the most recent, downscaled, rate constants. Following the execution of *N* new KMC steps, the algorithm is invoked again to check whether further downscaling is feasible. This is done over a different KMC time interval (*t*_*s*_, *t*_*f*_)*_m_*, where the subscript *m* counts the number of the downscaling attempts. The previous description corresponds to the first attempt, and the subscript *m* was omitted for brevity since m = 1. Finally, the cost and error are calculated in terms of the absolute downscale factor. Hence, from the second downscaling attempt onward, m > 1, we take into account any previous successful downscaling attempts, to retain the information regarding the overall speed gain and potential loss in accuracy.

#### 2.3.4 Additional Considerations in Decision Making

Apart from the weighting factors on cost and error, there are additional criteria put in place while evaluating whether the optimum downscale factor leads to an acceptable increase in the error. As mentioned earlier, the *k*th downscaled trajectory has the rate constants of the fast reaction reduced by . Practically, the first downscaled trajectory uses the same rate constants as the unscaled trajectory. In addition, the same random numbers are used for the generation of the slow reaction firings. What is different is that the fast reaction channels do not have their random number generator restored, and thus, the slow reaction firings in the unscaled trajectory do not occur in the same time instances as those of the “downscaled” one with *df* = 1. For reasons explained in detail in the SI, the slow reaction firing times have a distribution, with a certain mean and variance, and the latter depends on the time scale separation between the fast versus slow processes. To obtain a reference point for the IAT error norm, the generation of a trajectory with *df* = 1 is not only necessary but crucial as well, and its importance outweighs the additional computational expense for its generation.

By taking into account the reference IAT error norm and the IAT error norm of *df*_opt_, we impose the following additional criteria to prevent the overall error from increasing excessively:

a. absolute threshold: the IAT error norm that corresponds to *df*_opt_ should not exceed *e*_max_, taken as 0.05 in our benchmarks, and
b. relative threshold: the IAT error norm corresponds to *df*_opt_ should not increase by more than 2 orders of magnitude with respect to the IAT error norm of the downscaled trajectory generated using *df* = 1.

**Figure 3:**
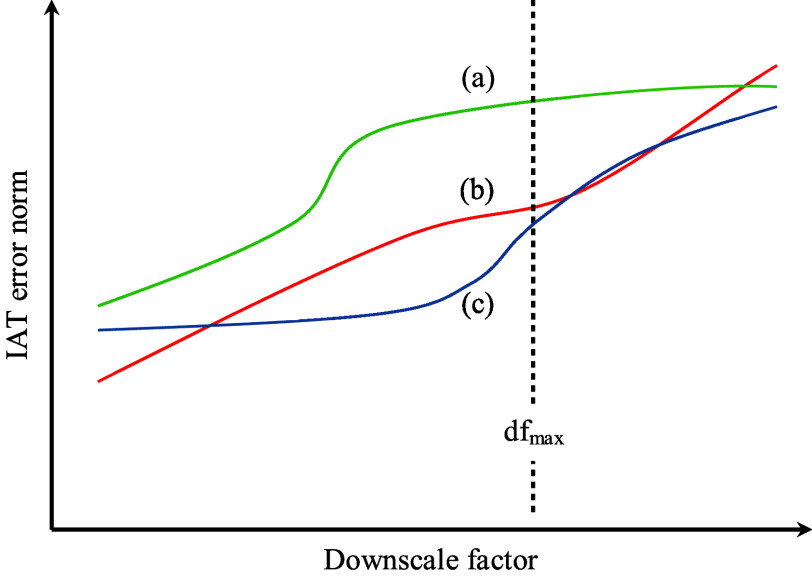
Qualitative error scaling with respect to the downscale factor for three fictitious slow reaction channels (a)–(c). Both axes are on logarithmic scale. The IAT error norm of reaction channel (c) appears saturated over the small downscale factors, whereas for reaction channel (a), the error norm is saturated over the large downscale factors, which are discarded anyway. Reaction channel (b) exhibits a more conventional scaling, for both small and large downscale factors. The vertical dashed line represents the maximum allowed downscale factor and data-points to its right are discarded.

A last criterion taken into account considers cases where the IAT error norm appears “saturated”, namely does not increase further with increasing downscale factor over the interval up to *df*_max_, as illustrated in Figure 3, curve (c). This phenomenon is an indication that the KMC time interval over which we attempt to do a downscaling is inappropriate for downscaling. To detect such cases, we impose a lower bound of 0.2 to the coefficient *q* of the equation  that is fitted to the IAT error norm data points for each slow reaction channel. We also note that we take into account data points up to *df*_max_, the vertical dashed line of Figure 3. As a result, the error curve of reaction channel (a) on Figure 3 does not suffer from error saturation, it is the scaling of reaction channel (c) that will cause the downscaling attempt to be aborted.

### 2.4 User-Tunable Parameters

We now summarize all the user-tunable parameters involved in our algorithm below and discuss their effect on performance.

- *N*: the number of KMC steps after which our downscaling algorithm is invoked. The larger *N* is, the less often our algorithm is called.
- *w = t*_*f*_ – *t*_*s*_: the width, in KMC time units, of the window over which the algorithm performs the downscaling evaluations. The larger *w* is, the more confidence we have on a downscaling if accepted, but at an increased computational cost. A good value for *w* is, generally, system dependent.
- *df*_b_: the base downscale factor used in the generation of multiple downscaled trajectories. Small values such as *df*_b_ = 2 would increase the total cost the downscaling attempts, whereas large values such as *df*_b_ = 20 could result in suboptimal choices for *df*_opt_ during the optimization procedure.
- *n*: the total number of downscaled trajectories generated inside the downscaling window including the one with *df* = 1. The generation of each subsequent trajectory is becoming cheaper, so large values on *n* do not affect performance. At the same time, generating too many downscaled trajectories may be redundant, because the trajectories in which the system is distorted are discarded.
- TSS_min_: the minimum time scale separation, quantified by the difference of the execution frequencies among the fast and slow reaction channels, in terms of orders of magnitude, that is required in order to proceed with the generation of the multiple downscaled trajectories. Larger values favor accuracy whereas smaller values allow for more downscaling and greater speedup.
- α, β: weight coefficients for the computational cost and error, respectively. Higher values of β favor the accuracy and therefore the *df*_opt_ is shifting to smaller values.
- *e*_max_: maximum absolute error threshold for any chosen downscale factor *df*_opt_. Smaller values may cause attempts to be rejected due to stricter accuracy requirements.
- *e*_inc_: maximum allowed absolute increase in orders of magnitude of the overall error norm. Smaller values limit the number of successful attempts as well as the overall reduction in the rate constants for the fast processes.

### 2.5 Summary of Algorithm and Flowchart

**Figure 4:**
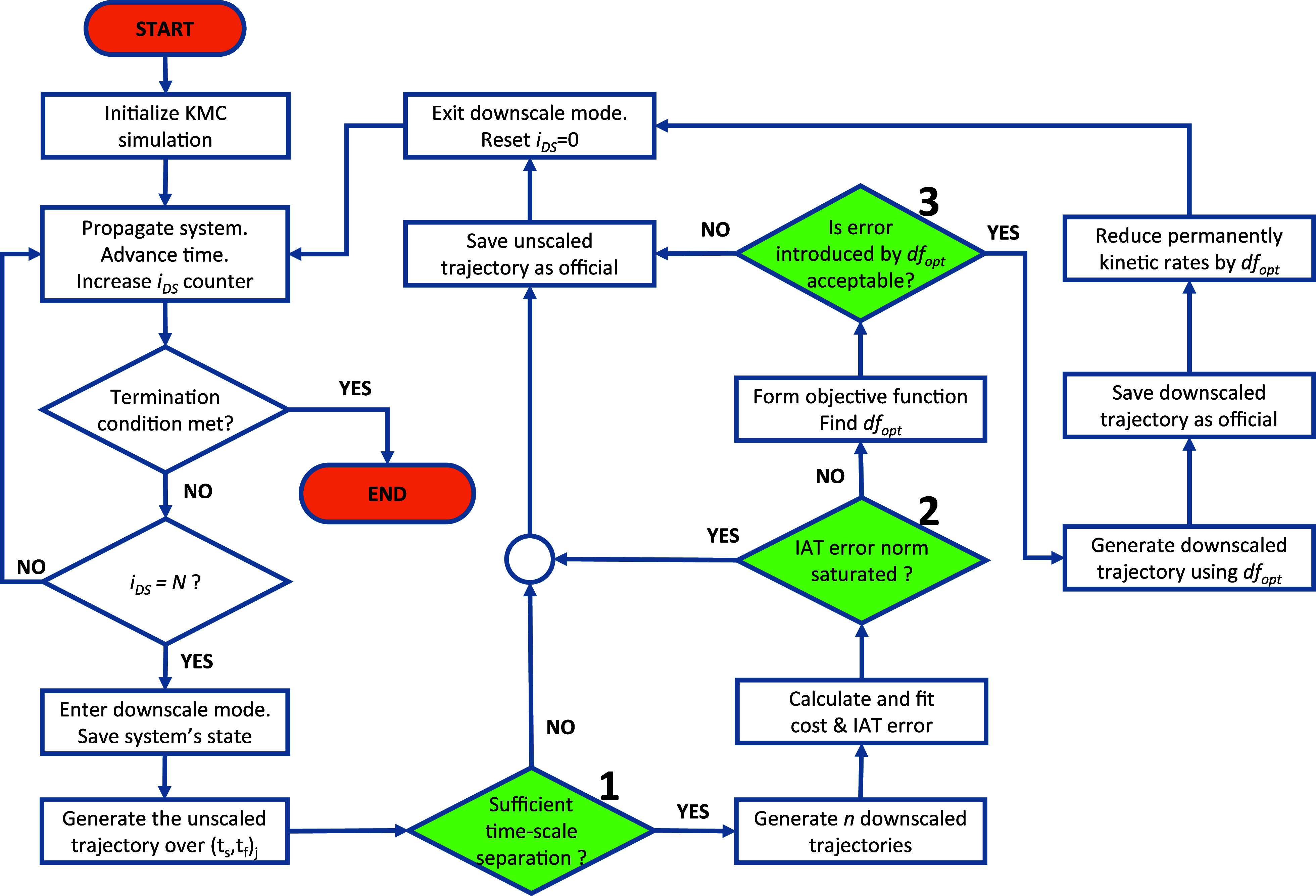
Flowchart of the proposed algorithm. The green shaded and numbered shapes represent the decision steps of the algorithm.

The procedures discussed in the previous sections are carried out concurrently with the essential tasks of a KMC run. To summarize our approach, we finally present a flowchart (Figure 4) and highlight the important components of our algorithm.

The main KMC loop that propagates the system forward in time is the same as in “traditional” KMC algorithms. The counter *i*_DS_ keeps track of the KMC steps executed for the purposes of invoking our algorithm when *i*_DS_ = *N,* at KMC time *t*_*s*_. The simulation enters the downscale mode, saves the state of the system, and generates the unscaled trajectory (Subsection 2.3.2). If there is sufficient separation between the fast and slow reaction in terms of execution frequency, then the downscaled trajectories are generated. Using the KMC steps executed and the firing times of all slow reaction events, we calculate the cost and IAT error and fit the appropriate equations to both. If the error checks on the scaling of the IAT error norm pass successfully, we form the objective function and find the optimum downscale factor. If its error is below the threshold, the corresponding trajectory is generated and officially accepted/registered, the rate constants are reduced, and the algorithm exits the downscale mode. Then the system is propagated in time using the reduced rate constants. In the case where a check fails, the downscaling attempt is aborted, the unscaled trajectory is registered as the official trajectory over the KMC time interval (*t*_*s*_, *t*_*f*_) and the KMC simulation continues as usual. The counter *i*_DS_ is reset and keeps track of the KMC steps to invoke the algorithm again when *i*_DS_ = *N*.

## 3 Computational Model, Results and Discussion

To validate our methodology and study its performance and efficiency, we apply the algorithm described above to an oscillatory reactive system inspired from biology. In the following, we first present our benchmark system along with its main features and identify the source of its temporal stiffness. We then present in detail the application of the downscaling algorithm on this system and show in practical terms the various algorithmic steps and decision criteria. In addition, we present and discuss cases where the downscaling is rejected, such as when the error is saturated. In the last subsection, we compare the results of the original, unscaled simulation with the downscaled one and discuss the validity of the methodology by presenting evidence that our algorithm did not distort the key features of the dynamics of the system. Matlab code implementing our methodology for the benchmark system is publicly available on Github: https://github.com/gsavva/KMC_downscaling.

### 3.1 Reaction Model

Our aim was to develop a generic on-the-fly rate scaling methodology rather than a system-specific one. For this reason, we carefully chose a benchmark system (a) with a good level of complexity, (b) with rich dynamic features, such as oscillations, which must not be distorted by the downscaling procedure, (c) in which the stiffness arises from pairs of reversible reactions, and (d) that is of practical interest to multidisciplinary fields. Regarding point (d), we also note that in biological systems, where the number of interacting molecules is typically low, stochastic effects may be significant for the evolution of the system.^32,33^ Taking into consideration the above points, we have chosen a biological oscillator that mimics the cell cycle as our benchmark system. The latter is a slightly modified version of the model introduced by Stamatakis and Mantzaris, as summarized in Table 3 in ref. (31) and includes nine reactions as presented in Table 1 below. For completeness, we note that the original model contains an additional reaction, X_2_ + Y → Y, which is herein neglected for simplicity without qualitatively altering the dynamics (the slow degradation of X_2_ of the original model still occurs as a two-step process, which proceeds first via dissociation to X).

**Table 1:** Reactions Included in Our Benchmark Model

No.	Reaction
1	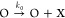
2	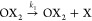
3	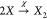
4	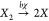
5	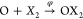
6	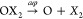
7	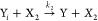
8	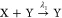
9	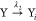

The reaction network of Table 1 involves the following six species: O, X, X_2_, OX_2_, Y_i_ and Y. Due to the system’s conservation laws O + OX_2_ = *O*_total_ and Y + Y_i_ = *Y*_total_, stemming from reactions 5, 6 and 7, 9, respectively, only the following four species are independent: O, X, X_2_ and Y. The populations of OX_2_ and Y_i_ are thus calculated using the conservation laws just presented. For more details on the physics of our benchmark model, we refer the interested readers to the original work.^31^ Briefly, we note that the oscillations on this system are caused by the autocatalytic action of species X, whose dimer, X_2_, exerts a positive feedback by further enhancing its own production, while Y, an active state of Y_i_, exerts negative feedback by degrading X via reaction 8 of Table 1. The nominal parameter set (including kinetic parameters and total species numbers) is provided in Table 2.

**Table 2:** Parameter values for the Benchmark Model^a^

Parameter	Value	Unit
*O*_total_	10	copy number
*Y*_total_	1035	copy number
*k*_0_	0.15	min^–1^
*k*_1_	50	min^–1^
*k*_2_	1.88 × 10^–3^	nM^–1^·min^–1^
φ	9.77·ζ	nM^–1^·min^–1^
χ	3.91·ζ	nM^–1^·min^–1^
ζ	100	dim/less
*a*	159.37	nM
*b*	5.31	nM
λ_1_	9.38 × 10^–3^	nM^–1^·min^–1^
λ_2_	0.01	min^–1^

a The parameter ζ controls the time scale separation or the stiffness of the system.

**Figure 5:**
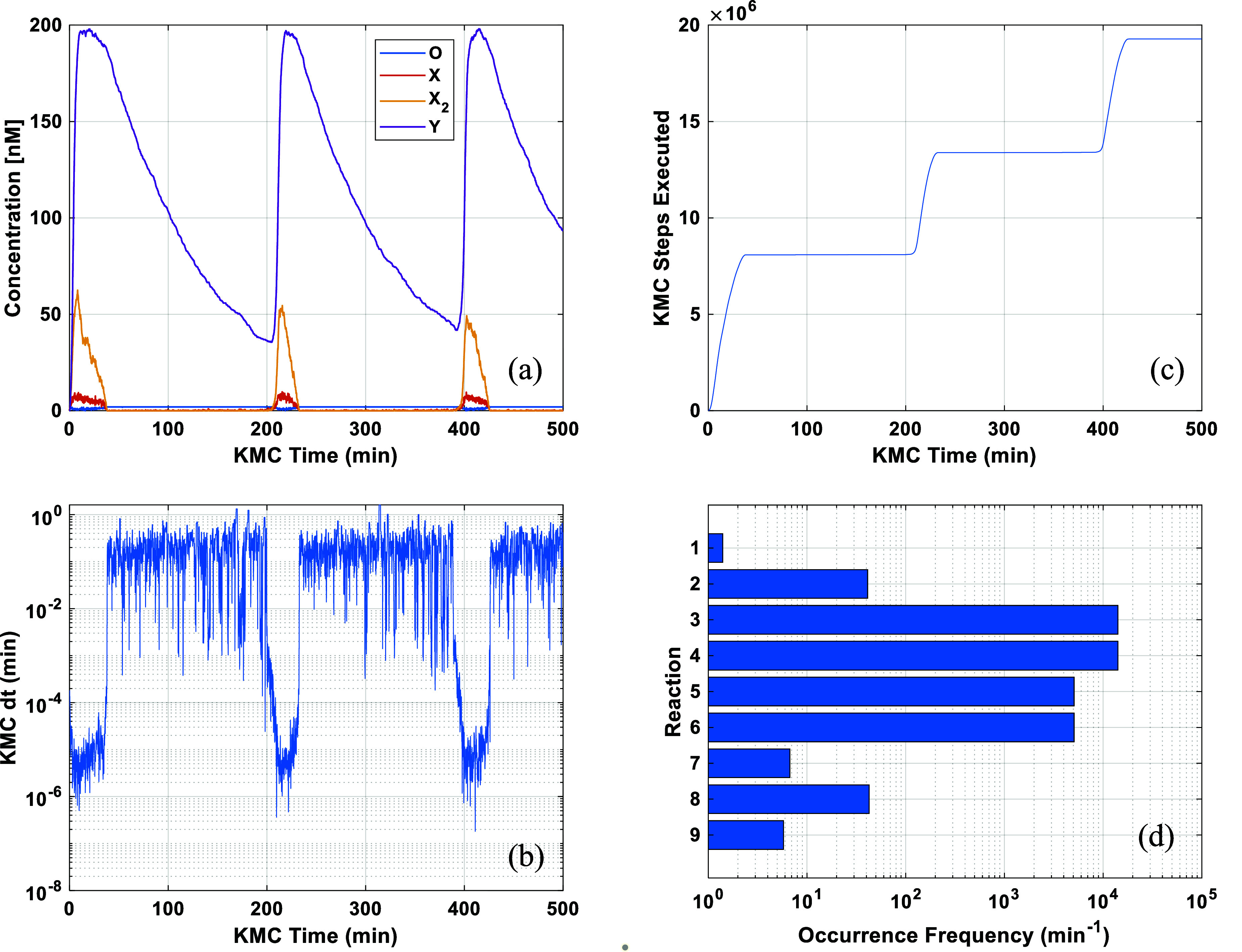
(a) Time evolution of key species of the benchmark system. (b) KMC time advancement with respect to KMC time. (c) KMC steps executed with respect to KMC time. (d) Overall execution frequencies of all the reactions in the network. Parameter values as in Table 2, except ζ = 10.

To demonstrate the main features of the benchmark system, we perform a simulation using the parameter values of Table 2, but with ζ = 10, with a final time of 500 min (in KMC time units). Figure 5a shows the time evolution of the concentrations of key species, highlighting the system’s oscillatory behavior with a period *T* ≈ 200 min. For most of the time, the concentrations of species X and X_2_, and thus, the propensities of reactions 3–6 are small or exactly zero. The KMC time advancement with respect to KMC time (Figure 5b) reveals that the system has fast- and slow-propagating regions. Figure 5c also shows that the computational cost, quantified by the number of KMC steps executed, varies during the course of the simulation. More specifically, as illustrated in Figure 5c, 8 × 10^6^ KMC steps are executed to propagate the system until KMC time 40, whereas only a tiny fraction thereof (13 × 10^3^) is needed to further propagate the system until KMC time 200. Combining the visual information on the panels (a), (b) and (c) of Figure 5, we conclude that the slow-propagating regions occur when the concentrations of species X and X_2_ become nonzero and the concentration of Y increases rapidly. Lastly, the overall execution frequency of all reactions in our network is illustrated in Figure 5d. Clearly, reactions 3–4 and 5–6 are quasi-equilibrated, and they dominate the computational expense. More specifically, reactions 3–6 are executed at least 2 orders of magnitude more frequently than the next most frequently executed reactions 2 and 8, and take up 99.5% of the total executed KMC steps, making the system “stiff”.

A closer look at the reaction network and Figure 5, reveals that once reactions 3–6 are quasi-equilibrated, they no longer contribute any transient features to the dynamics of the system. Based on the above, along with the criteria introduced in the beginning of this section, the chosen benchmark system is ideal for our downscaling methodology and could help us draw conclusions on the generality of the latter.

### 3.2 Application of the Downscaling Algorithm

We proceed to applying the developed methodology to the benchmark system and discussing our results on cases where downscaling is successful, versus cases where it is aborted because an error-related check fails. As already discussed in Section 2.3.1, for the algorithm to scale down the rate constants of the fast reactions, we provide information about the expected execution frequency of the reactions in our network and indicate the reactions whose rates may undergo reduction, i.e., the fast reactions 3–6, in our case. Thus, the parameters allowed to be downscaled are χ and φ only.

The algorithm is invoked after a total number of *N* = 10^5^ KMC steps are executed. We have chosen our base downscale factor as *df*_b_ = 5, hence the rate parameters χ and φ are reduced in powers of *df*_b_ such as 5, 25, 125, etc. A total of *n* = 9 downscaled trajectories are generated in each attempt; the first trajectory has *df = df*_*b*_^*0*^ = 1, and the maximum reduction of the rate constants is done by *df = df*_b_^*8*^ = 390625. The minimum required time scale separation, TSS_min_, in the execution frequencies between the fast and the slow reactions is set to 2 orders of magnitude. Thus, the algorithm will only attempt a downscaling if the fast reactions, especially 5–6, are at least 10^2^ = 100 times faster than the fastest among the slow reactions, reactions 2 and 8 as shown in Figure 5d. The weights on the cost and error are set as α = 1 and β = 2, respectively, since we aim for better accuracy. Finally, we allow a maximum IAT error of 0.05 and a maximum increase in the error by 2 orders of magnitude. The latter is feasible since we obtain a reference error point by generating the trajectory with *df* = 1. The values of all user-defined parameters used in our simulations are summarized in Table 3. Lastly, for the parameter ζ that appears in the rate parameters in Table 2 we have chosen the value of ζ = 100 unless otherwise stated.

**Table 3:** User-Defined Parameters of the Developed Algorithm as Discussed in Section 2 and Summarized in Section 2.5

Parameter	Value	Units
*N*	10^5^	KMC steps
*w*_min_ = *t_f_* – *t_s_*	10	KMC time units
*w*_max_ = *t_f_* – *t_s_*	15	KMC time units
*df*_b_	5	-
n	9	-
TSS_min_	2	Orders of magnitude
α	1	-
β	2	-
*e*_max_	0.05	-
e_incr_	2	Orders of magnitude

**Figure 6:**
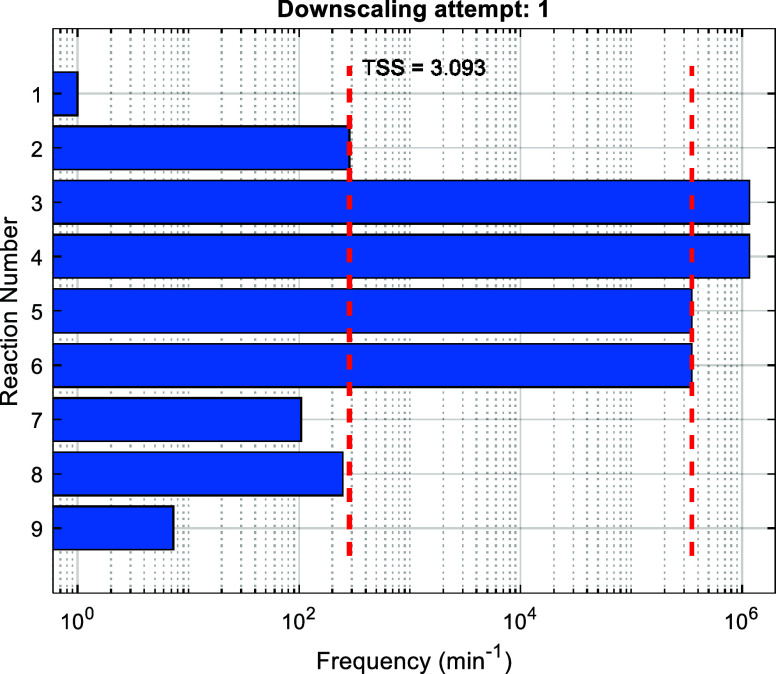
Execution frequencies of all reaction channels of the unscaled trajectory over the first downscaling interval (1.503, 10.503).

Upon initialization of the KMC simulation, the time is set to zero and the species populations are set to zero apart from OX_2_ and Y_*i*_ that are initialized to *O*_total_ and *Y*_total_, respectively (see Table 2). The simulation runs as usual for *N* = 10^5^ and reaches a time of *t*_KMC_ = 1.503 min, at which point the downscaling algorithm is invoked. The state of the system is saved, and the width of the current downscaling window is chosen as *w* = 10 min, which is the minimum width we impose. Over the KMC time the interval (1.503, 10.503) the algorithm generates the unscaled trajectory of the system and calculates the reaction execution frequencies (as illustrated in Figure 6). The TSS is found to be 3.093, which is sufficient based on the user-defined TSS_min_, therefore, the algorithm proceeds in restoring the state of the system back to *t* = 1.503 min and generating the *n* = 9 downscaled trajectories. Once the downscaled trajectories are obtained, the algorithm moves on to the evaluation and decision-making stage.

**Figure 7:**
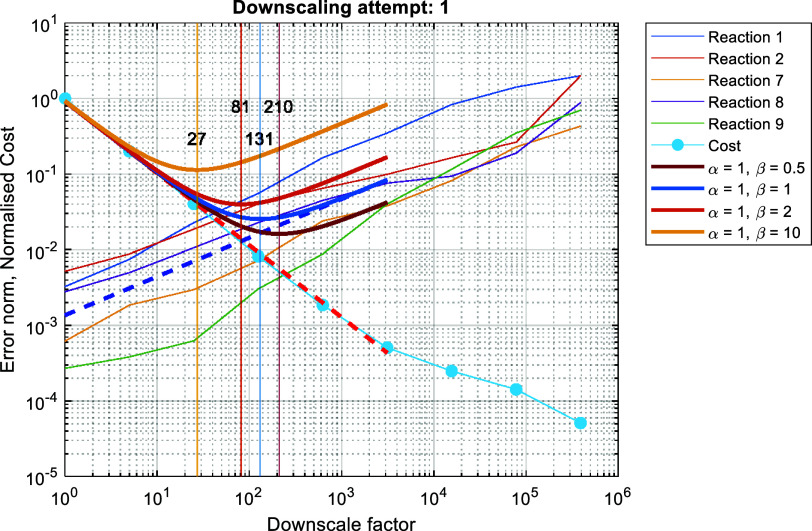
Cost and IAT error norm with respect to the downscale factor. The red dashed line is the fit of the cost. The blue dashed line is the aggregated fit on the IAT error. The colored bold lines are the objective functions for different weights on cost and error as listed in the legend. The colored vertical lines and the overlaid numbers correspond to the minimum of the different objective functions, matched by color.

Using the collected data, such as KMC steps executed and the occurrence times of all slow reaction firings, the cost and IAT error norm are calculated as discussed in Section 2.3.3. Both quantities are plotted against the downscale factor in Figure 7. The additional error check concludes that the IAT error norm for each of the slow reaction channels scales as expected, i.e., none of those error norms is saturated. Before forming the objective function, we fit the cost and IAT error data points to obtain the analytical expressions for the cost, *C*(*df*), and error, *E*(*df*), respectively, as shown in Figure 7 by the red and blue bold dashed lines. More specifically, for the fit on the IAT error, we use all the error data-points from all slow reactions and perform a single fitting to obtain *E(df)*. Then, the objective function is formed using eq 10 and the weights as reported in Table 3. The minimum of the objective function (bold dark orange curve in Figure 7) suggests that the downscale factor *df* = 81.3 strikes the ideal balance between cost and accuracy. The latter downscale factor does not violate any of the additional error checks, as the absolute error incurred is 0.013, below the *e*_max_ = 0.05, and the overall error increase remains below the 2 orders of magnitude since the average error increases by a factor of 10. For comparison purposes, we use additional pairs of weight factors for the cost and error, to illustrate how the weights affect the objective function and thus the optimum downscale factor chosen. The additional objective function curves are illustrated with bold continuous curves in Figure 7 and the corresponding weights are as listed in the legend.

**Figure 8:**
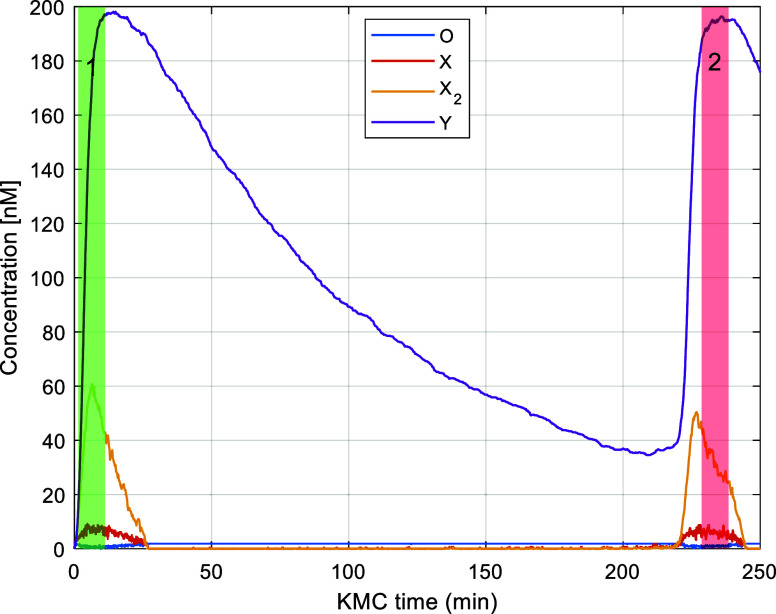
Trajectory of the system obtained with downscaling algorithm active. The green vertical band represents the first downscaling attempt, which was successful whereas the red vertical band the second attempt, which was aborted.

The decision-making procedure identified the optimum downscale factor as *df*_opt_ = 81.3. Then, for the final time, the state of the system is restored back to *t*_KMC_ = 1.503 min, to generate the final downscaled trajectory of the system using *df*_opt_. The latter trajectory is registered as “official”, the species concentration samples are saved along the samples collected outside any downscaling attempt, the rates χ and φ are reduced permanently by a factor of *df*_opt_, and finally, the algorithm exits the downscale mode. In addition, the counter, *i*_DS_, that triggers the downscaling attempts is reset. From that point onward, the system is propagated as usual with the reduced rate constants. Once another *N* = 10^5^ KMC steps are executed, the algorithm is invoked again to check for downscaling. This happens at KMC time *t*_KMC_ = 228.7. The second downscaling window (*t*_*s*_, *t*_*f*_)_2_ is determined as (228.7, 238.7)_2_ as illustrated in Figure 8. In the second attempt, the time scale separation between the fast and slow reactions is less than the user-defined minimum and thus, the attempt is aborted. We would like to note that an aborted attempt, due to insufficient time scale separation (decision block #1 in flowchart of Figure 4), has negligible overhead because the already generated unscaled trajectory is used to fill the KMC time interval (*t*_*s*_, *t*_*f*_)_*j*_ for any *j* the attempt is aborted.

**Figure 9:**
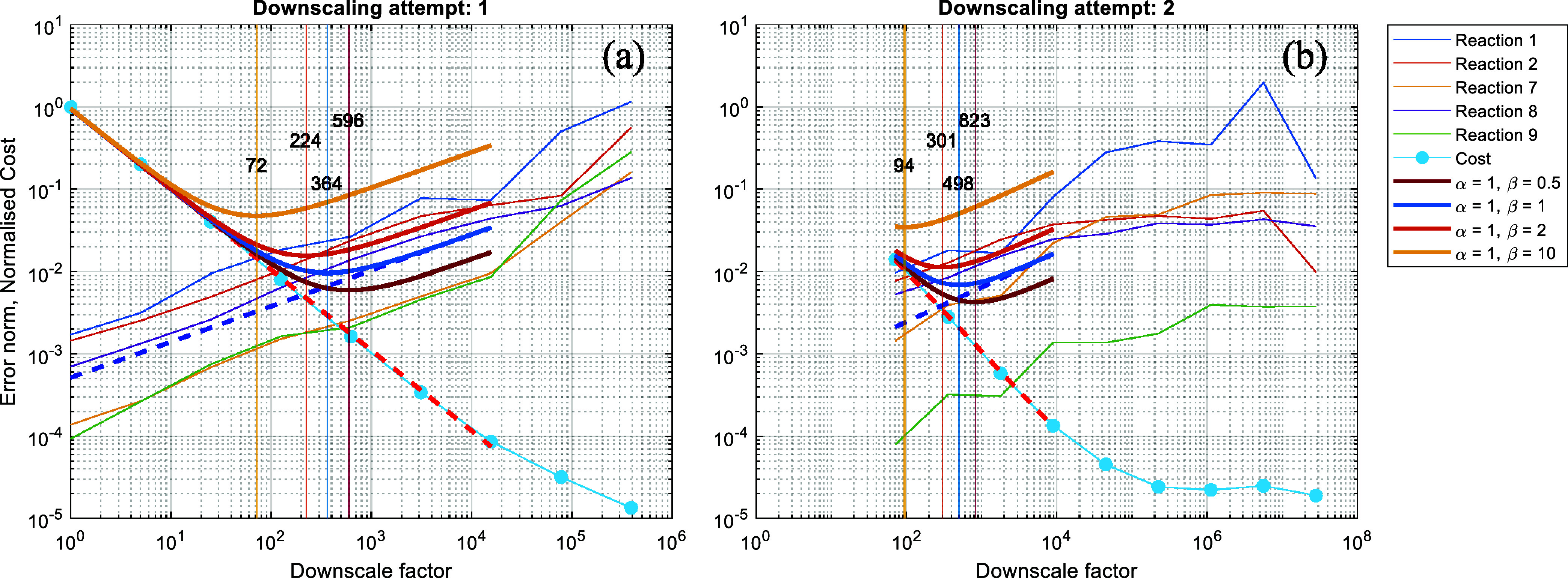
Scaling of the error norm and normalized cost with the corresponding objective functions for the two accepted downscaling attempts. On the right panel, the curves start at 72.1 which was the chosen *df*_opt_ during the 1st attempt.

**Figure 10:**
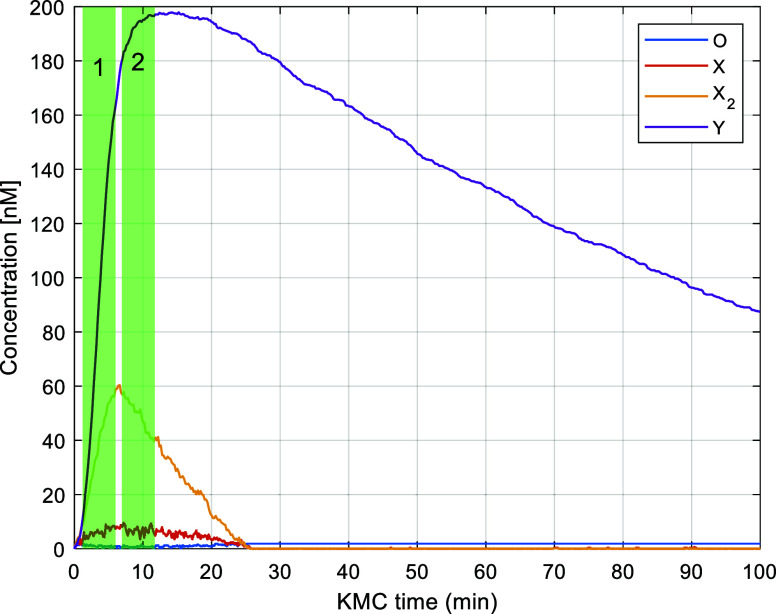
Location, in terms of KMC time, of the two accepted downscaling attempts denoted by the two vertical green bands.

In the run just discussed, the tunable parameters were chosen such that the first downscaling attempt reduces the execution frequency of the fast processes up to the point that no more downscaling is permissible. To illustrate how the algorithm takes into account previous successful attempts, we performed a second test run, choosing a different value for the parameter ζ so that the time scale separation is larger and therefore, more reduction on the corresponding rate constants is possible. Thus, the simulation results presented below are obtained using ζ = 1000, *w*_min_ = 5, and β = 10. These parameters were chosen such that the computational cost of the first downscaling attempt is reduced and the first successful downscaling would allow the second one to be accepted as well; thus, the first downscaling is less aggressive favoring accuracy rather than cost-reduction. The first attempt is successful, identifying *df*_opt_ = 72.1, and the relevant error and cost curves along with various objectives functions are plotted in Figure 9a. The second attempt is also successful. The methodology deems that a further reduction by a factor of 1.3 is acceptable so that the overall downscale factor becomes 94, as shown in Figure 9b. The time ranges over which the two successful attempts took place are illustrated in Figure 10.

Based on the results of Figure 9, it is worth discussing a few important points. First, in all attempts, the calculation of the cost takes into account any previous accepted attempts. This is crucial to propagate information about the actual cost of the downscaled trajectories generated in subsequent downscaling attempts. Second, for every downscaling attempt, the downscaled trajectories are obtained by reducing the *current* rate constants by powers of the user-provided *df*_b_. Since the *current* rate constants are used, the reduction procedure is done in a relative manner during a downscaling attempt. For example, during the second attempt, the rate constants that have already been reduced by a factor of 72.1 (the *df*_opt_ of the first attempt), are further reduced by 5, 25, 125, etc. However, the decision procedures are performed using the absolute downscale factor as illustrated in Figure 9b.

### 3.3 Results and Validation

We use the parameters of Table 2 with ζ = 10 and run our benchmark model twice until a final time of 5 × 10^6^ KMC minutes. For the first run, the downscaling methodology is disabled and therefore the simulation is executed from start to finish using the initial rate constants. For the second run, the downscaling algorithm is enabled. The user-defined parameters values are as listed in Table 3 except for the weight on the error, β, which was chosen as β = 1, favoring cost reduction. In the latter run, following the procedure described above, the optimum downscale factor was identified as *df*_opt_ = 125 right from the first downscaling attempt and no further reductions occurred. The run with the original rate constants reached the final KMC time in 66 h of wall time, while the run in which the rates were reduced completed in just 90 min. The developed methodology thus accelerated the second run by 44 times, achieving a significant reduction in the total computational cost.

The acceleration factor just noted is probably on the conservative side, since the reason for choosing the parameter value ζ = 10 for our benchmarks was to be able to run simulations within a reasonable amount of time (66 h). Choosing a larger value for ζ would have resulted in greater overall speedup, however running the original system would have been intractable without spending computational resources for an unreasonably high amount of time. In fact, the time scale separation between slow and fast kinetics in actual systems encountered in biology can be much larger than 10. For instance, in a gene expression kinetic model of the LacZ and LacY proteins,^34^ which has been used extensively for benchmarking approximate acceleration methods for stochastic chemical kinetics,^6,9,35,36^ the rate constants span over almost 7 orders of magnitude, and the propensities range over at least 6 orders of magnitude.^9^ Another notable example is the intracellular viral infection model,^37^ used as benchmark by Haseltine and Rawlings,^12^ in which the rate constants span over 8 orders of magnitude, thus making the system stiff. Thus, our methodology could lead to considerable acceleration factors for systems with such significant time-scale separation. For instance, we estimated that the original simulation with ζ = 100 would have a runtime between 27 and 28 days, while the one with the downscaling enabled takes only 3.2 h (parameters as Table 3, *w*_min_ = 5, *df*_b_ = 12, β = 1, *df*_opt_ = 288). Using the former estimate of 27–28 days, along with the actual runtime of 3.2 h we obtained for the reduced system, the speed-up gain ranges from 200× to 207×. In principle, the speedup may be much higher than 200× in cases where the time scale separation is much larger than 2 to 3 orders of magnitude.

**Figure 11:**
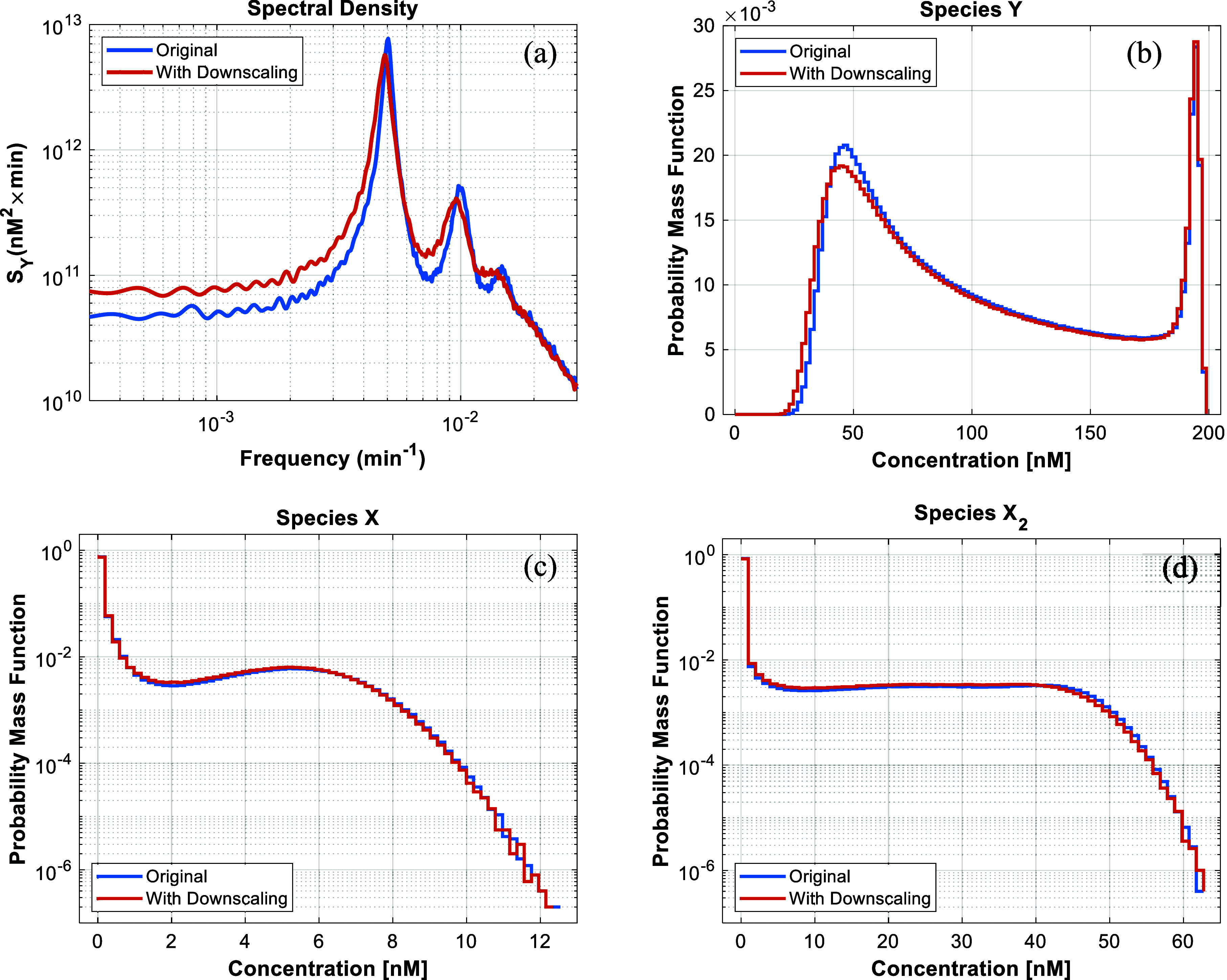
Comparisons between the original and downscaled system, both run for 5 × 10^6^ KMC minutes, of the spectral density of species Y (a), and the probability density function of species Y (b), species X (c), and species X_2_ (d). Parameter values as in Table 2 with ζ = 10.

The main reason we opted for a tractable original system is to facilitate the validation. The stochastic nature of the KMC method makes it impossible to directly compare trajectories (concentration versus time), to demonstrate the equivalence of the original and the downscaled versions of the system. However, a comparison can be made on the basis of average features. In our benchmark model, the oscillatory behavior is the main feature, motivating an examination of whether the downscaling incurred a change on the dominant oscillation frequency. To this end, we calculate the autocorrelation function of the concentration of species Y and subsequently the power spectral density via a cosine transformation, as explained in section IV of ref. (31). In Figure 11a, we plot the spectral density of the trajectory of species Y for both the original and downscaled system. Clearly, the dominant frequency of *f*_0_ = 5 × 10^–3^ min, which corresponds to a period of *T* ≈ 200 min (cf. Figure 5a), is exhibited by the reduced system, thereby validating the developed method. In addition, the probability mass functions of the concentrations of species Y, X and X_2_, as illustrated in Figure 11b–d, respectively, show that the downscaling algorithm incurred minimal error.

Following the above discussion on obtaining the dominant frequency of our benchmark model, it important to also discuss the higher frequency features simulated. For a trajectory with a single periodic component, such as in our benchmark system, one may obtain peaks in the spectral density on the dominant frequency as well as on integer multiples thereof that correspond to the component’s harmonic frequencies. Indeed, in Figure 11a, the curve for the system with the original rate constants exhibits three peaks that correspond to the frequencies of *f*_0_, 2 × *f*_0_ and 3 × *f*_0_. However, in the spectral density curve of the system with downscaling, the frequency 3 × *f*_0_ is suppressed. This effect is expected because the downscaling procedure is known to alter the high frequency dynamics due to the slowing-down of the fast events, which accelerates significantly the computational simulation.

## 4 Summary and Conclusions

In this study, we developed a methodology to reduce on the fly the rate constants of very frequent and quasi-equilibrated processes in well-mixed chemical systems. At specific intervals during the course of the simulation, our algorithm generates sets of multiple trajectories, each trajectory featuring rate constants that are reduced by a different factor, and collects data on the computational cost and the error introduced. Then, an optimization problem is solved to identify the downscale or reduction factor that achieves the best balance between accelerating the simulation with the least error. Our algorithm includes some well-defined, user-provided parameters to tune its performance, depending on the user’s preference on favoring speed or accuracy. To assess the performance of our algorithm, we have chosen as our benchmark a biology-inspired chemical oscillator.^31^

This study aims to fill the gap in methods that tackle the time scale disparity on well-mixed systems, especially regarding the quantification of error introduced because of the reduction of the rate constants of the fast processes. Our method is based on the same underlying concept that underpins the AS-KMC methodology;^21^ however, AS-KMC keeps track of states visited during the simulation and processes that “connect” these states to detect the existence of superbasins. While this is in principle a general and powerful approach, simulations of complex systems whose accessible state space is large may be impractical and thus we resort to a simpler approach which detects fast and quasi-equilibrated events. Moreover, AS-KMC lacks an algorithmic procedure by which information on the error is fed-back into the downscaling procedure. In the algorithm developed, the reduction of the rate constants of fast reaction channels is data-driven, and the optimum downscale factor is chosen by solving an optimization problem, aiming to balance the introduction of error and the reduction of the computational cost. Most importantly, our algorithm provides metrics on the error introduced, and by specifying some user-tunable parameters, one may favor accuracy or computational efficiency according to their needs.

The application of our algorithm on the benchmark system demonstrated reductions of the simulation runtime from 66 h to 90 min, providing an acceleration factor of 44×. The reported acceleration factor probably underestimates the capabilities of the methodology since we have chosen the parameters of the benchmark system such that we may obtain the original run in a reasonable amount of time (in order to perform detailed comparisons in our benchmarks). We also estimate that an acceleration factor of 200× is easily obtained. In principle, much larger acceleration factors may be obtained if the time scale separation between the fast and slow reactions is greater, also given that in more realistic physical systems the rate constants may span over 8 orders of magnitude.

## Data Availability

Matlab code implementing the methodology developed for the benchmark system of this study is publicly available in https://github.com/gsavva/KMC_downscaling.

## Supporting Information Available

Detailed discussion of (1) the background (established) methods underpinning our approach and (2) computational results on the source of the nonzero interarrival time error norm while using the Modified Next Reaction method in combination with the Common Random Number method (PDF)
