# Supplementary material for: Tackling the Temporal Stiffness of Kinetic Monte Carlo Simulations of Well-Mixed Chemical Systems via On-the-Fly Scaling and Cost-Error Optimization
Source: J Phys Chem A. 2025 Feb 5;129(6):1726–40. doi: 10.1021/acs.jpca.4c05963 (PMC11831668; doi:10.1021/acs.jpca.4c05963)
Supplement: Supplementary file 1 — jp4c05963_si_001.pdf [file jp4c05963_si_001.pdf]

Tackling the Temporal Stiffness of Kinetic Monte Carlo Simulations of Well-Mixed Chemical Systems via On-the-Fly Scaling and Cost-Error Optimization

Giannis D. Savva[†] and Michail Stamatakis[‡,*]

Thomas Young Centre and Department of Chemical Engineering, University College London, Roberts Building, Torrington Place, London WC1E 7JE, UK

[†] Current address: Laboratory of theory and simulation of materials, EPFL STI IMX THEOS, Station 9, 1015 Lausanne, Switzerland

[‡] Current address: Inorganic Chemistry Laboratory, Department of Chemistry, University of Oxford, South Parks Road, Oxford, OX1 3QR, United Kingdom

[*] e-mail: michail.stamatakis@chem.ox.ac.uk

SUPPLEMENTARY MATERIAL

1. Methodological Background

The stochastic simulation algorithm (SSA), originally derived by Gillespie, is an exact method of simulating numerically the stochastic time evolution of spatially homogeneous mixtures of interacting species, often termed as "well-mixed systems". Since the introduction of two variants of this method by Gillespie, additional approaches and further improvements have been proposed. In the following, we present briefly the relevant KMC methods and highlight features thereof that are further used in our work. In addition, we introduce two widely used methods in parameter sensitivity analysis, namely the Common Reaction Number and Common Reaction Path which are also used in our work.

1.1. First Reaction Method (FRM)

The First Reaction Method is one of the two procedures proposed by Gillespie.[1] It is based on the intuitive idea that, given a set of feasible reactions along with their interarrival firing times, the reaction to occur next is the one with the smallest waiting time. Therefore, for every possible reaction i, a tentative reaction time τ_i is generated via:

$$\tau_i = -\frac{\ln(r_i)}{a_i} \tag{1}$$

where r_i is a uniformly distributed random number in the unit interval and a_i is the propensity function

of reaction i. The reaction μ that occurs next and its interarrival time τ_μ are both determined by finding the minimum among all the reaction waiting times τ_i:

$$\tau_\mu = \min_i \left(\tau_i\right) \quad (2)$$

Then, the KMC clock is advanced by τ_μ, and the reaction μ is executed by updating the molecular populations accordingly. New tentative reaction times are generated using equation (1) and the procedure described above is repeated.

For every iteration, the First Reaction Method requires N new random numbers, where N is the number of reactions in the reaction network. Regenerating all tentative reaction times might not be necessary, however. If the execution of reaction μ does not change the propensity function a_j of reaction j through the modification of the populations of any of the involved species, then the waiting time τ_j is still valid and could be used in the determination of the next reaction to occur. Although useful, the computational "trick" just described might not be applicable to complex and strongly coupled reaction networks, in the sense that, for such networks, the execution of any reaction event would affect the majority of the other reactions, and no computational savings would be obtained by the above "trick". For such cases, one would still be forced to generate a significant number of uniformly distributed random deviates, which could lead to computational inefficiencies.

1.2. Next Reaction Method (NRM)

Identifying the drawbacks of the First Reaction Method as using many random numbers per KMC step and having a computational cost that scales linearly with respect to the number of reactions, Gibson and Bruck developed the Next Reaction Method where they introduced a number of new features.[2] First, the method operates with absolute times, denoted by t_i, instead of relative waiting times, τ_i. Second, the algorithm uses exactly one random number per reaction execution, excluding the initialization step during which the random numbers used are as many as the reactions of the system modelled. Third, on the implementation level, the method makes use of a dependency graph and indexed priority queues to speed up the reaction scheduling operations.

The NRM builds upon the ideas of the FRM with some modifications. At the beginning, all reactions are assigned an occurrence time t_i using the expression (1) above. The minimum among all the occurrence times, t_μ, is obtained and the KMC clock is set to t_μ, $t_{KMC} = t_\mu$. Reaction μ is executed, the molecular populations are updated and the propensities α_i of the affected reactions are updated as well. Then, for the reaction just occurred ($i = \mu$), a new random number, r, is generated and the new occurrence time is obtained as:

$$t_i^{new} = t_{KMC} - \frac{ln(r)}{a_i^{new}} \tag{3}$$

For all other affected reactions ($i \neq \mu$), the occurrence times are *adjusted* without the generation of new random numbers according to the equation:

$$t_i^{new} = t_{KMC} + \frac{a_i^{old}}{a_i^{new}} \cdot \left(t_i^{old} - t_{KMC}\right) \tag{4}$$

where a_i^{old} and a_i^{new} are the propensity functions of reaction i before and after the execution of reaction μ respectively, and t_i^{old} is the occurrence time of reaction i before the execution of reaction μ. In practice, the occurrence time is decreased or increased, with respect to the current KMC time, t_{KMC}, so that it reflects the increase or decrease of the propensity function. No new random numbers are used at this stage.

During a KMC run, it may happen that a propensity function a_i becomes zero. For as long as the propensity is zero, the corresponding occurrence time, t_i, should be set to ∞, i.e., the reaction will not occur at any finite time. In such cases, the equation (4) presented above requires modification.[2] The correct time adjustment transformation when a propensity goes to zero and then ceases to be zero is:

$$t_i^{new} = t_2 + \frac{a_i^{old}}{a_i^{new}} \cdot \left(t_i^{old} - t_1\right) \tag{5}$$

where t_1 is the KMC time when a_i first became zero, t_2 is the KMC time when a_i ceased to be zero, a_i^{old} is the last pre-zero propensity and a_i^{new} is the first post-zero propensity.

Especially in biological systems, where species participate in low populations, propensities might become zero and then reach non-zero levels again quite frequently. Implementing the NRM in a way to handle zero propensities would require significant bookkeeping of pre- and post-zero quantities thereby complicating the implementation significantly.

1.3. Modified Next Reaction Method

By changing the representation of reaction times and viewing them as firing times of independent, unit rate Poisson processes, Anderson[3] developed a modified version of the Next Reaction Method of Gibson and Bruck.[2] The advantages of the new method that are relevant to our work are (**a**) the randomness in the model being uncoupled from the state of the system and (**b**) its generality and its inherent ability to handle zero propensities without any change to the core algorithm. The latter naturally leads to a simpler implementation even for complex systems with multiple species and

reactions. For these reasons, we used the Modified Next Reaction Method (Mod-NRM) in our work, the algorithm of which is outlined in the following. For mathematical derivations and physical meanings of the quantities defined, the reader is referred to the original work[3] and more specifically to section III thereof.

At the beginning of the KMC run, set the KMC time, t_{KMC}, to zero. For every reaction i, calculate the propensity functions a_i, initialize the "internal times" T_i to zero and set

$$P_i = -\ln(r_i) \tag{6}$$

where r_i is a uniformly distributed random number in the range (0,1). Then, calculate the firing times of every reaction i as:

$$\tau_i = \frac{P_i - T_i}{a_i} \tag{7}$$

Find the minimum among all τ_i, $\tau_\mu = \min_i(\tau_i)$, advance the KMC time, t_{KMC}, by τ_μ and update the molecular populations according to reaction μ. For every reaction i, update the "internal times" T_i as:

$$T_i \leftarrow T_i + a_i \tau_\mu \tag{8}$$

For the reaction that just occurred ($i = \mu$), generate a new random number, r, and update P_μ as follows:

$$P_\mu \leftarrow P_\mu - \ln(r) \tag{9}$$

Lastly, recalculate all the propensities a_i, or just the affected ones for increased efficiency. New waiting times are generated using equation (7) and the steps following equation (7) are repeated until a step-based or time-based termination criterion is met.

We note again that the Mod-NRM consumes exactly one random number per reaction execution via equation (9), excluding the initialization step. As compared to the NRM, the Mod-NRM operates with time advances, τ_μ, instead of absolute times and, more importantly, zero propensities do not require a different transformation. Elaborating on the latter point, we note that when a propensity a_i becomes zero, the corresponding interarrival firing time, as per equation (7), becomes infinity. However, the internal time, T_i, remains unaffected since it is "updated" to the same value as per (8). P_i is also unaffected since the reaction i will never occur because its propensity is zero. When a_i becomes non-zero, a finite value (not infinity) is generated for τ_i via (7), using values for P_i and T_i that are not infinity.

1.4. Random Time Change (RTC) method

Working towards efficient methods for computing parameter sensitivities in biochemical networks and changing the representation of reaction times, Rathinam and co-workers developed the Random Time Change (RTC) method.[4] Their method is mostly similar to the Mod-NRM of Anderson[3] apart from a conceptually significant difference. In the Mod-NRM[3] and, in fact, in any other KMC method described so far,[1-2] all the random numbers consumed during the initialization step, via the relations (1) or (6), and during the simulation via (1), (3) or (9), are drawn from a single random number stream. In the RTC method, N independent, parallel random number streams are used, where N is the number of reactions in the system studied, corresponding to one random number stream per reaction channel. Therefore, when using equations (6) and (9), a different random number stream is used for each value of the index *i*, i.e., for each different reaction channel.

1.5. Common Random Number (CRN) method

In the context of KMC simulations, one wishes to collect uncorrelated samples of the state of the simulated process or system, so a high-quality random number generator is used. Not unexpectedly, reusing the same random numbers has generally been avoided in production runs. In sensitivity analysis studies, however, using on purpose the same random numbers across different runs, hence the term "common", has been an easy and well-known method to reduce the variance by introducing dependence, viz. non-zero covariance, between the results being compared.[5] By using the same random numbers among different runs, one is able to rule out stochasticity-induced changes to the system's trajectories. By combining the latter approach with appropriate mathematical methods such as finite differences, one may quantify how the system's trajectory changes if one parameter is perturbed from c_0 to c_0+h. In other words, the CRN method makes it possible to obtain quantitative metrics of the sensitivity of a stochastically simulated system on certain parameters by comparing appropriately the reference and the perturbed results.

For sensitivity analysis purposes, when combined with any KMC method, the CRN method is used as follows:[4] a random seed, *rs*, is used to initialize the random number generator and for a given parameter c_0, a solution trajectory of the system studied is obtained. Next, a second trajectory is generated by performing another KMC run in which (a) the random number generator is initialized using the same random seed, *rs*, that was used for the first run, and (b) the rate parameter is perturbed by h and its value is changed from c_0 to c_0+h. Since the same random numbers are used in the two runs, thereby eliminating the stochastic component that would differentiate the results, any differences between the two trajectories corresponding to the parameters c_0 and c_0+h respectively, are attributed exclusively to the perturbation h, added to the original rate parameter c_0.

Even though the idea of the CRNs is not a novel concept, the combination of a KMC method, and specifically the RTC, along with the CRN enabled the development of yet another method described below.

1.6. Common Reaction Path (CRP) method

The Random Time Change (RTC) method described in section 1.4 differs from all other KMC methods because it uses as many random number streams as the reactions in the systems for the generation of tentative firing times. This unique feature of the RTC algorithm in conjunction with the CRN method has been instrumental in the development of the Common Reaction Path (CRP) method by the same authors[4] as an efficient approach for computing parameter sensitivities. Without getting into the details about its performance as compared to other methods, we outline the idea of the CRP method and make the connection with our work later on.

We have already mentioned that the RTC uses one random number stream per reaction channel and that the CRN method reuses the same sequence of random numbers for multiple runs by initializing the random number generator with the same seed. Combining these two features results in the CRP, an algorithm in which the *internal* jump times of each reaction channel remain unaffected among the perturbed and unperturbed simulations. Note that this invariance of the internal jump times requires both that each channel has its own random number stream and that the same random numbers are used. On the contrary, if using the CRN method along with any KMC method other than the RTC, such invariance would not be achieved, i.e., the internal jump times of the perturbed simulation would be different from those of the unperturbed one. This happens because, even though the random numbers are drawn from a single stream, there is no guarantee that they are used in the same order in the perturbed versus in the unperturbed run.[4]

2. Convergence of Slow Reaction Firing Times wrt. Time scale Separation

A major difference of the Modified Next Reaction Method (Mod-NRM) by Anderson[3] as compared to the First Reaction Method by Gillespie[1] is that the former consumes just one random number per KMC step, excluding the initialization stage of the simulation. In Mod-NRM, the reaction channels affected by the executed reaction have their interarrival times adjusted in order to reflect the change of their propensities. Due to the change in the representation of firing times in the Mod-NRM, the "loss of memory"-property is not invoked,[3] i.e., the firing times of the reactions are affected by the previous history and not just by the state of the system, i.e., the molecular populations.

The coupling between the fast and slow reactions, and the use of internal times T_i, combined with not using the "loss of memory"-property causes the occurrence times of the downscaled trajectory with $df = 1$ to differ from the unscaled trajectory, even though the kinetic rates are the same and the same random numbers are used to generate the firing times of the slow reactions. The latter is also the source of the non-zero error norm we have shown for $df = 1$ (Figure 7 on main text). This error norm depends on the time scale separation between the fast and slow reactions.

Here, we demonstrate computationally the convergence of the firing times with respect to the time scale separation. Equivalently, the error norm gets smaller as the time scale separation becomes larger. To do so, we use the following "toy-model":

$$A \xrightarrow{k_1} B$$

$$B \xrightarrow{k_2} A$$

$$B \xrightarrow{k_3} C$$

We use the Mod-NRM to propagate the above system in time. We start with an initial population of $A_0 = 50$ molecules and $B_0 = 30$ molecules. For the kinetic rates, we have chosen

$$k_1 = 5\times10^{N}\ \mathrm{s}^{-1}$$

$$k_2 = 8\times10^{N}\ \mathrm{s}^{-1}$$

$$k_3 = 1\ \mathrm{s}^{-1}$$

where the exponent N controls the time scale separation. We use two different random number streams: one for the fast and reversible pair of reactions A ↔ B and another for the slow reaction B → C. The first random number stream is initialized randomly, whereas the second is initialized with a specific seed and is kept the same for all the runs.

To collect statistics on the occurrence time of the first B → C reaction, we run the system described above, with $N = 1$, for $R = 5000$ times. The distribution of the firing times of interest is plotted on Figure 1. It is easy to see that, despite the fact that the same random number is used to generate the firing time of the first B → C transition, the latter firing time is not exactly the same on every run.

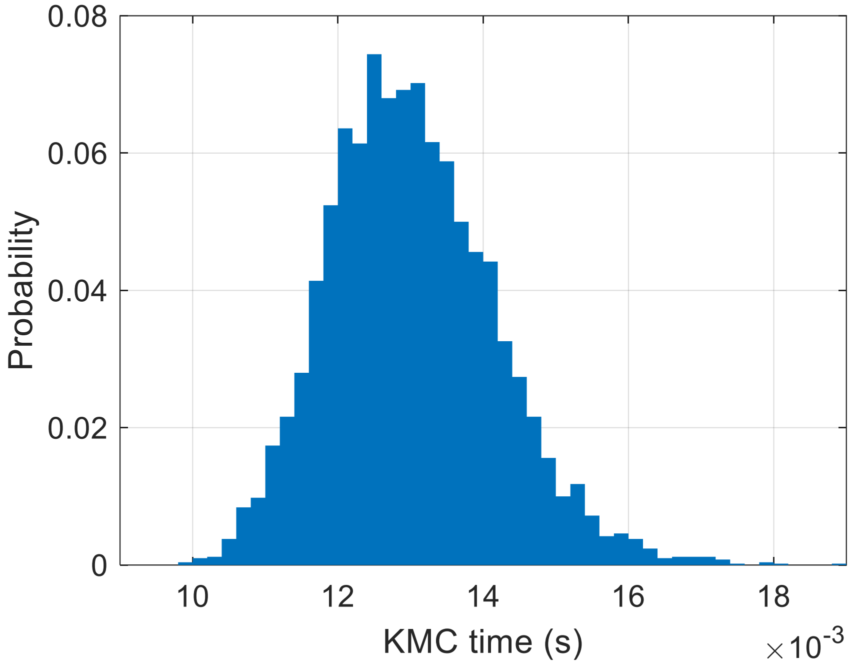

Figure 1: Distribution of the firing times of the first B $\longrightarrow$ C reaction. The samples were collected from R = 5000 repetitions of the KMC simulation.

To investigate the dependence of the distribution on the time scale separation, we run the same simulation as above for different values of the exponent N. We collect samples from R = 5000 KMC runs for each value of N. Our results are presented on Figure 2.

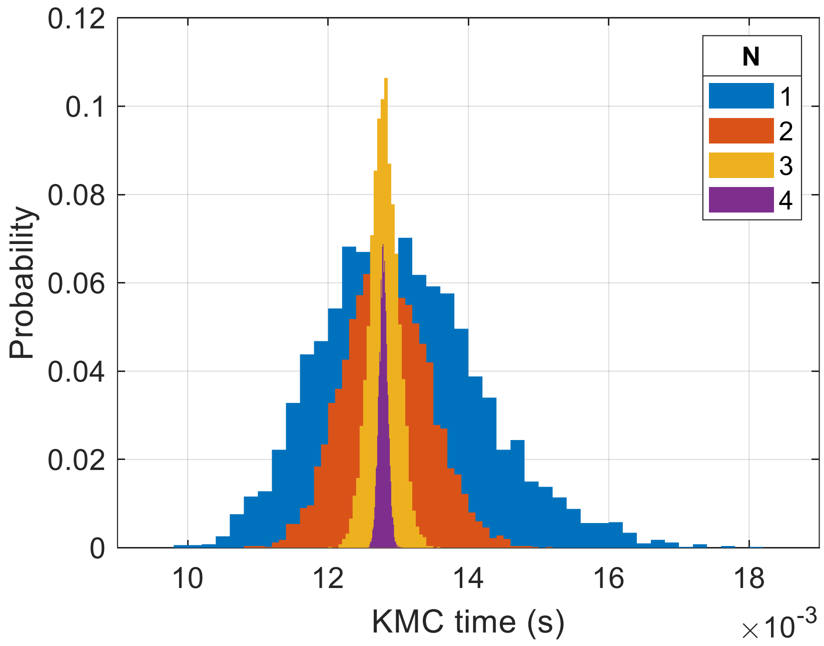

Figure 2: Distributions of the firing time of the first B $\longrightarrow$ C reaction.

From the above results, we observe that the distribution becomes narrower as the time scale separation between the fast and slow reactions increases, equivalently, the standard deviation of the distributions decreases, whereas the mean of the distributions seems unchanged. Our results suggest that the firing times of the slow reaction, here B → C, would converge to a single value, had the A ↔ B reversible isomerization was infinitely fast. However, in our toy-model here, and our benchmark model as introduced in the main text, the fast reversible pairs of reactions are not infinitely fast. The latter along with the with-memory Mod-NRM gives rise to the non-zero error norm, as defined in the main text, when the same system is simulated under the same conditions and the same random numbers for the slow reactions.

References

(1) Gillespie, D. T. A General Method for Numerically Simulating the Stochastic Time Evolution of Coupled Chemical Reactions. *J. Comp. Phys.* **1976,** *22*, 403-434.

(2) Gibson, M. A.; Bruck, J. Efficient Exact Stochastic Simulation of Chemical Systems with Many Species and Many Channels. *J. Phys. Chem. A* **2000,** *104*, 1876-1889.

(3) Anderson, D. F. A Modified Next Reaction Method for Simulating Chemical Systems with Time Dependent Propensities and Delays. *J. Chem. Phys.* **2007,** *127*, 214107.

(4) Rathinam, M.; Sheppard, P. W.; Khammash, M. Efficient Computation of Parameter Sensitivities of Discrete Stochastic Chemical Reaction Networks. *J. Chem. Phys.* **2010,** *132*.

(5) Glasserman, P.; Yao, D. D. Some Guidelines and Guarantees for Common Random Numbers. *Manage Sci.* **1992,** *38*, 884-908.
